# Glucocorticoid-Responsive Cold Agglutinin Disease in a Patient with Rheumatoid Arthritis

**DOI:** 10.1155/2015/823563

**Published:** 2015-08-06

**Authors:** Kyoko Honne, Takao Nagashima, Masahiro Iwamoto, Toyomi Kamesaki, Seiji Minota

**Affiliations:** ^1^Division of Rheumatology and Clinical Immunology, Department of Medicine, Jichi Medical University, Yakushiji 3311-1, Shimotsuke, Tochigi 329-0498, Japan; ^2^Center for Community Medicine, Jichi Medical University, Yakushiji 3311-1, Shimotsuke, Tochigi 329-0498, Japan

## Abstract

A 57-year-old man with rheumatoid arthritis developed severe anemia during treatment with adalimumab plus methotrexate. Cold agglutinin disease was diagnosed because haptoglobin was undetectable, cold agglutinin was positive (1 : 2048), and the direct Coombs test was positive (only to complement). Although the cold agglutinin titer was normalized (1 : 64) after treatment with prednisolone (0.7 mg/kg/day for two weeks), the patient's hemoglobin did not increase above 8 g/dL. When cold agglutinins were reexamined using red blood cells suspended in bovine serum albumin, the titer was still positive at 1 : 1024. Furthermore, the cold agglutinin had a wide thermal amplitude, since the titer was 1 : 16 at 30°C and 1 : 1 at 37°C. This suggested that the cold agglutinin would show pathogenicity even at body temperature. After the dose of prednisolone was increased to 1 mg/kg/day, the patient's hemoglobin rapidly returned to the normal range. The thermal amplitude test using red blood cells suspended in bovine serum albumin is more sensitive than the standard test for detecting pathogenic cold agglutinins.

## 1. Introduction

Cold agglutinin disease (CAD) is an immune-mediated hemolytic anemia caused by IgM autoantibodies (cold agglutinins) that exhibit maximum reactivity with human red blood cells (RBC) at low temperatures of 0–4°C [1]. CAD accounts for 8 to 25% of autoimmune hemolytic anemia [1–3]. It is classified as primary (idiopathic) or secondary, and secondary CAD has two main causes that are malignancy or infections [1, 3].

CAD is rarely associated with connective tissue diseases [2, 4] and there have been few case reports of this disease in patients with rheumatoid arthritis (RA) [5–7]. Although CAD is an autoimmune disorder, glucocorticoid treatment is generally ineffective. This provides a sharp contrast with warm autoimmune hemolytic anemia, which generally shows a good response to glucocorticoids [1, 4]. Here we report a patient with RA who developed acute-onset CAD and showed a good response to glucocorticoid treatment.

## 2. Case Report

**Figure 1:**
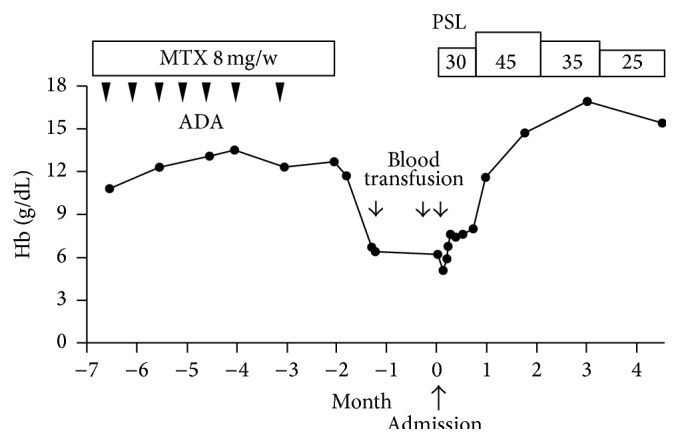
Clinical course of the patient and hemoglobin levels. ADA: adalimumab; Hb: hemoglobin; MTX: methotrexate; PSL: prednisolone (mg/day).

A 57-year-old man had an 8-year history of RA and had been treated with methotrexate for 6 years. Biweekly injection of adalimumab had been started 6 months earlier to suppress disease activity. He showed a good response to adalimumab and achieved clinical remission. After seven doses, adalimumab and methotrexate were both suspended due to upper respiratory tract infection. One month later, he suddenly developed severe anemia that was unresponsive to blood transfusion (Figure 1). He was then referred to our hospital for assessment.

On admission, laboratory tests revealed a white blood cell count of 11,300/*μ*L, RBC count of 191 × 10^4^/*μ*L, hemoglobin of 6.2 g/dL, mean corpuscular volume of 95 fL, platelet count of 48.2 × 10^4^/*μ*L, and reticulocyte count of 18.3%. There were spherocytes in the peripheral blood smear, but he had no family history of hereditary spherocytosis. Total bilirubin was 2.3 mg/dL (normal ≤ 1.5), direct bilirubin was 0.1 mg/dL, lactate dehydrogenase was 351 U/L (normal ≤ 216), and C-reactive protein was 7.6 mg/dL. Rheumatoid factor and anticyclic citrullinated peptide antibody were positive, but antinuclear antibody and anti-double-stranded DNA antibody were negative. Complement components C3 and C4 were 90 mg/dL (normal: 86–160) and 12 mg/dL (normal: 17–45), respectively. Serum immunoelectrophoresis revealed no monoclonal proteins. Haptoglobin was below the detection limit. The direct Coombs test was negative for IgG but was positive for complement. Cold agglutinin was positive at 1 : 2048 (normal < 1 : 128). Serology for* Mycoplasma pneumoniae* was negative, and examination of anti-Epstein-Barr virus antibodies suggested a postinfectious state. There had been no episodes of hemoglobinuria, acrocyanosis after exposure to cold, or Raynaud's phenomenon. Because the patient's hemoglobin decreased further to 4.5 g/dL, two units of RBC were transfused and treatment was started with prednisolone (PSL) at 30 mg/day (0.7 mg/kg) under the diagnosis of CAD. Although cold agglutinin became negative (1 : 64) after treatment with PSL for two weeks, his hemoglobin did not recover above 8 g/dL. Haptoglobin was still below the detection limit and complement component C3 decreased further to 67 mg/dL. We reexamined cold agglutinins using RBC suspended in bovine serum albumin, instead of RBC suspended in saline. The test revealed that the cold agglutinin titer was 1 : 1024 at 4°C, 1 : 16 at 30°C, and 1 : 1 at 37°C. This suggested that cold agglutinin was still present and that hemolysis was continuing because the agglutinin was reactive at temperatures above 30°C.

The dose of PSL was increased from 30 mg/day to 45 mg/day (1 mg/kg) and the patient's hemoglobin increased rapidly to 14.7 g/dL after one month, while complement levels returned to the normal range after three months. Readministration of methotrexate with tapering of PSL did not lead to the recurrence of hemolysis. A lymphoproliferative disorder has not emerged after more than four years.

## 3. Discussion

In this patient, CAD developed during the course of RA and showed a good response to glucocorticoid therapy. Because upper respiratory tract infection preceded the sudden decrease of hemoglobin, an unknown viral infection was the most likely cause of CAD. However, acute-onset CAD associated with infection is usually self-limiting and resolves within several weeks [3, 8, 9], while hemolysis continued for more than two months in our patient, which is a relatively long duration. Since CAD is an immune-mediated disorder, an association with RA is possible [4, 6]. Adalimumab could also be considered as the cause, because etanercept (another antitumor necrosis factor agent) has been reported to induce CAD [5].

Detection of cold agglutinins by using RBC suspended in bovine serum albumin is reported to be more sensitive than by using RBC suspended in saline [10, 11]. Reduction of the zeta potential has been proposed as one of the mechanisms by which bovine serum albumin increases sensitivity for cold agglutinins [12]. In the present patient, although cold agglutinin became negative in the standard test after treatment with PSL for two weeks, the titer was still high (1 : 1024) when measured using bovine serum albumin. Furthermore, it became evident that the thermal amplitude (the highest temperature at which the autoantibody binds to RBC) was wide and agglutination occurred even at 30°C to 37°C. The pathogenicity of a cold agglutinin depends on its thermal amplitude rather than the agglutinin titer [1, 9]. If a cold agglutinin is reactive at temperatures above 30°C, it can cause hemolysis* in vivo*.

Primary chronic CAD is generally refractory to pharmacotherapy, including glucocorticoids [13, 14], although some cases of glucocorticoid-responsive CAD have been reported [13, 15, 16]. When CAD responds to glucocorticoid therapy, the cold agglutinin titer tends to be relatively low and the agglutinin has a wide thermal range [13]. In the present patient, the cold agglutinin also had such features. Patients with these findings are sometimes classified as having “low-titer” CAD. However, the definition of “low-titer” is not completely clear, although a titer of less than 1000 was considered to be low in the original report [13]. Patients with acute hemolysis and polyclonal cold agglutinins may also respond to glucocorticoid therapy and this is a feature of infection-related CAD [3, 8]. In fact, efficacy of glucocorticoids has been reported in some patients who developed CAD associated with* Mycoplasma* infection [14, 17, 18].

We reviewed nine patients who had CAD associated with connective tissue diseases and were reported in the English literature (Table 1) [5, 6, 19–24]. The cold agglutinin titer was less than 1000 in six of these nine patients. Most of them had severe anemia. Various treatments were reported and all of the patients except one responded. The treatments included hydroxychloroquine, low-dose/high-dose PSL with or without immunosuppressants, and rituximab. No treatment was given to one patient with polymyalgia rheumatica, who was also the only patient with a monoclonal cold agglutinin. Therefore, CAD associated with connective tissue diseases may respond to glucocorticoid therapy if a monoclonal cold agglutinin is not detected. Primary chronic CAD is usually resistant to glucocorticoid therapy and monoclonal IgM is found in 90% of these patients [3].

In conclusion, high-dose glucocorticoid therapy was effective for acute-onset CAD in a patient with RA. The thermal amplitude test using RBC suspended in bovine serum albumin is more sensitive than the standard test for detecting pathogenic cold agglutinins.

## Figures and Tables

**Table 1:** Characteristics of the patients who had CAD associated with connective tissue diseases.

Case number	Diagnosis	Age, sex	Raynaud's phenomenon or acrocyanosis	Splenomegaly	CA titer	Monoclonal IgM	Hb (g/dL)	Treatment
1	SSc	60, F	Yes	Yes	1 : 256^†^	No	6.1	Pulse + mPSL (60 mg)

2	SS	78, F	Yes	n.d.	1 : 64	n.d.	5.0	PSL (10 mg), dopamine, pyridoxal, PGE_1_, and systemic warming

3	SLE	55, F	n.d.	n.d.	1 : 512	n.d.	7.9	Pulse with PSL (30 mg) relapse: PSL (40 mg), DFPP, CyA, and RTX

4	SLE	27, F	No	No	1 : 7000	No	6.7	Prednisone (40 mg) and plaquenil

5	SLE	34, F	No	Yes	1 : 4096	n.d.	6.0	PSL (60 mg)

6	RA	52, F	n.d.	n.d.	1 : 512	No	9.1	PSL (1 mg/kg), CyA, and RTX

7	RA	89, F	Yes	No	1 : 320	No	31%^‡^	Hydroxychloroquine

8	PMR	60, M	Yes	n.d.	1 : 256^†^	Yes	8.0	Avoiding cold

Present case	RA	57, M	No	No	1 : 2048	No	6.2	PSL (1 mg/kg)

CA, cold agglutinin; CAD, cold agglutinin disease; CyA, cyclosporin; DFPP, double-filtration plasmapheresis; Hb, hemoglobin; Ht, hematocrit; mPSL, methylprednisolone; n.d., not described; PGE_1_, prostaglandin E_1_; PMR, polymyalgia rheumatica; PSL, prednisolone; RA, rheumatoid arthritis; RTX, rituximab; SLE, systemic lupus erythematosus; SS, Sjögren's syndrome; SSc, systemic sclerosis. ^†^The titer was measured at room temperature. ^‡^Hematocrit.
